# Supplementary material for: Asynchronous magnetic resonance elastography: Shear wave speed reconstruction using noise correlation of incoherent waves
Source: Magn Reson Med. 2022 Oct 27;89(3):990–1001. doi: 10.1002/mrm.29502 (PMC9792433; doi:10.1002/mrm.29502)
Supplement: Supplementary file 1 — DATA S1 MATLAB code used to generate the numerical simulations of Figure 1 [file MRM-89-990-s002.zip › k-Wave/helpfiles/example_ivp_setting_initial_gradient.html]

Setting An Initial Pressure Gradient Example (k-Wave)


# Setting An Initial Pressure Gradient Example

This example demonstrates how to set an initial pressure gradient using `kspaceSecondOrder`. It builds on the Comparison Of Modelling Functions Example.

- Open the file in the MATLAB Editor
- Run the file in MATLAB

## Contents

- Defining the source properties

## Defining the source properties

The function `kspaceSecondOrder` allows initial conditions for both the initial pressure and its temporal gradient to be defined. The initial conditions are defined as a matrix of numeric values the same size as the computational grid. The initial pressure is assigned to `source.p0`, while the initial pressure gradient is assigned to `source.dp0dt`.

In this example, `makeDisc` is used to create an initial source distribution of two small discs with different diameters. This is first assigned to the `p0` field of the `source` structure, then to the `dp0dt` field, and then to both fields. The optional input `'MeshPlot'` is set to `true` to allow a 3D visualisation of the pressure field. If `'PlotFrames'` is set to `true` the individual frames are spawned in new figures. A plot of the pressure field at two different values of t is shown below.
